# Quality of life of patients living with psoriasis: a qualitative study

**DOI:** 10.1186/s12895-020-00116-9

**Published:** 2020-12-10

**Authors:** Silmara Meneguin, Natália Aparecida de Godoy, Camila Fernandes Pollo, Hélio Amante Miot, Cesar de Oliveira

**Affiliations:** 1 Department of Nursing, Botucatu Medical School, Paulista State University, São Paulo, Brazil; 2 Department of Dermatology, Botucatu Medical School, Paulista State University, São Paulo, Brazil; 3 Department of Epidemiology & Public health, University College London, London, UK

**Keywords:** Psoriasis, Quality of life, Dermatology, Nursing

## Abstract

**Background:**

Psoriasis is a multifactorial inflammatory disease prevalent in dermatology. We aimed to understand the perceptions of patients living with psoriasis in relation to their quality of life and to identify aspects to improve it.

**Methods:**

This is qualitative research carried out in a dermatology outpatient clinic of the São Paulo State University (UNESP) medical school, Botucatu, Brazil, with 81 psoriasis patients. The interviews were transcribed and analysed using the Discourse of the Collective Subject method (DCS).

**Results:**

Quality of life was linked to well-being, happiness, leisure, good food and financial stability. However, disease symptoms, social and clothing restrictions, impairment of professional activities and the absence of a cure, negatively influenced their perceptions. Suggestions for improvements included an increase of public awareness, stress reduction, disease acceptance and multidisciplinary care.

**Conclusion:**

The meanings of quality of life revealed by the participants are subjective, multidimensional, linked to moments experienced by them and to the health-disease process. Public health policies promoting reduction in social stigma and stress as well as multidisciplinary approaches towards care can contribute to improvements of QoL in psoriasis.

**Supplementary Information:**

The online version contains supplementary material available at 10.1186/s12895-020-00116-9.

## Background

Psoriasis is a chronic inflammatory dermatosis characterized by the appearance of erythematous-desquamative, symmetric plaques with white-silver desquamation, which may present extracutaneous manifestations, such as arthritis and uveitis [1]. In Brazil, its prevalence is estimated between 1.1 and 1.5% and represents between 4.0 and 4.8% of dermatological consultations [1–4]. The social and psychological impacts caused by psoriasis are usually underestimated by health professionals [5].

As with other immune-mediated complex diseases, there is no definitive cure for psoriasis, and the current available treatment only decreases disease activity and improves symptoms [3]. Psoriasis is a chronic and recurrent disease that affects visible areas and joints and impacts on quality of life [6].

The World Health Organization (WHO) defines quality of life as the individual’s perception of their position in life, in the context of the culture and value system in which they live, in relation to their goals, expectations, standards and concerns [7]. However, three aspects are common in all definitions: subjectivity, dimensionality and bipolarity [8].

Based on the assumption that dermatoses can affect self-image and have a great potential for triggering processes that affect self-esteem, they may contribute to feelings such as anxiety, sadness or even depressive symptoms. Therefore, the perception of quality of life is considered a critical measure in dermatology [9]. In this sense, quality of life evaluation has become an indicator used to guide health care practices and help in the definition of strategies of public policies, since the determinants and constraints of the health-disease process are multifactorial and complex.

At present, there is a need to refine quality of life measures for patients with psoriasis, in order to identify issues that are important to them, such as disease stigma and prejudice, among others. Often, these are not included in the quality of life instruments available in the literature.

Therefore, the present investigation aimed to understand the perceptions of patients living with psoriasis in relation to their quality of life and to identify potential aspects to improve it. This study will explore the individual experiences of psoriasis patients and, ultimately, to contribute to the knowledge in a poorly investigated area of ​​dermatology.

## Methods

A descriptive exploratory study with a qualitative approach was undertaken. The research was conducted with patients with psoriasis at a dermatology outpatient clinic of the São Paulo State University (UNESP) medical school, Botucatu, Brazil.

The following eligibility criteria was adopted: patients diagnosed with psoriasis, aged 18 years or over, of both sexes, who consented to participate. Participants who reported being emotionally vulnerable to either participate or continue the interview were excluded. A non-probabilistic sample comprised of 81 patients who attended the outpatient dermatology clinic of the São Paulo State University (UNESP) medical school, Botucatu, from December 2017 to May 2018. This sample corresponded to the total number of patients attending the clinic in the period of data collection. There were neither refusals nor interrupted interviews. The interviews were conducted by two medical professors and an undergraduate student without any kind of link with the participants. The transcripts of the interviews could not be returned to the participants for comments.

Data were collected through semi-structured face-to-face interviews, including sociodemographic data and the following guiding questions: What do you understand by quality of life? In your opinion, does psoriasis interfere with your quality of life? If yes, in what aspects? In your perception, how could your quality of life be improved in relation to psoriasis? [see Additional file 1].

The interviews were individual, and audio graphed and conducted in a private setting, before the medical consultation by one of the researchers fully trained in the technique used in the data collection. The interview time was on average 20 min.

The data were analysed using the Discourse of the Collective Subject (DCS) method, based on the theory of social representations [10]. The DCS seeks to respond to the challenge of self-expression of thought or collective opinion, respecting the dual qualitative and quantitative condition of both.

Considering that the materialized thought about the form of the discourse is a form of qualitative variable, that is, it is a product to be later qualified. However, this collective thought is also a quantitative variable insofar as it expresses the opinions shared by individuals [11], and does not use the theory saturation criteria to interrupt an interview. In our study, all patients who attended the outpatient dermatology clinic and agreed to participate in the interview during the data collection period were interviewed. The choice of this type of approach was based on the option of understanding the meanings, motives, aspirations, attitudes and beliefs supported in the nature of the study object [12].

The methodological steps of this technique, from obtaining the interviews to the synthesis of the speeches, included: reading the set of testimonies collected in the interviews; reading the response to each question, marking the selected key expressions; identification of the central ideas of each response; analysis of all key expressions and central ideas, grouping the similarities into homogeneous sets; identification and naming of the central idea of the homogeneous whole, which will be a synthesis of the central ideas of each discourse; construction of the discourses of the collective subject after the identification of the central ideas and key expressions that have named the discourses of the collective subject [12].

The project was approved by the ethics committee of the São Paulo State University (UNESP), Sao Paulo (protocol number 2.392.601). All participants signed a statement of informed consent. In this consent, the researchers described all steps to ensure confidentiality i.e. the researcher’s agreement to handle, store, and share research data to ensure that information obtained from and about research participants remains confidential.

## Results

Regarding the characterization of the 81 participants, most were male (54%), married/living with a partner (70%) and completed elementary education (60%). The mean age of the patients was 50 years and the onset of the disease was at the age of 36. For 61% of the sample, the monthly family income varied between $322 and $879.

From the transcription analysis of the interviews, the central ideas and the key expressions were identified, and the discourses of the three themes emerged from the guiding questions. The following topics are presented with their respective central ideas and patients’ DCS.

### Theme 1 - meaning of quality of life

Central ideas:

A. Well-being
B. Solidarity
C. Financial stability
D. Good nutrition
E. Do not have stress and concerns
F. Be happy
G. Having leisure and practicing physical activity
H. Being employed

DCS - *In my view, quality of life is the ability to live well, being able to go out and walk, to have a good house, leisure and a life ... it is my welfare, to be able to live happily!! I also feel that it is important to feel physically and mentally well ... so I try to eat healthy and exercise. I drink a lot of water and try not to stress about everyday things. I think that a good relationship with everyone is important, besides being honest, having a job that is stable, and to like what you do. Having a close family and faith make all the difference. I do not need a lot of money to have a good life, no!!! For me, quality of life is also having friends, but that is different from person to person, because after we go through some things in life, everything else become less important. If you understand people, quality of life improves, regardless of financial aspects, I think being a bit more human simplifies things because everyone is living very busy lives, so I think if we stop for a moment and think about others, things will improve for everyone. Quality of life is not having disease, or if you have one, is to have the means to treat it, to be able to buy medicine, because many people cannot afford it. And it's not enough just to come to the hospital, but you have to follow the doctor’s recommendations at home too.*

### Theme 2 - impact of psoriasis on quality of life

Central ideas:

A. Symptoms of the disease (pain and itching)
B. Behaviour (shame)
C. Increased stress
D. Frustration and suffering
E. Link inseparable from treatment
F. Commitment to professional activities
G. Stigma of disease
H. Contagiousness
I. Restriction on clothing

DCS - *It interferes with everything for me. Psoriasis hurts, especially when it is aggravated, and when it itches when I am around others. When I scratch too much, and it's a bother, with the plaques coming off. It's so itchy and addictive, and once you put your hand on it, it does not stop itching anymore. I think when I am inside my house it does not matter, but on the street it is complicated because we are ashamed. I am ashamed of everything, I try to avoid places that have a lot of people, and if someone stares at me, I find myself scratching. You do not have much freedom ... when you are swimming you cannot be comfortable because some people understand, others do not. They think they will catch it. At first, it was difficult because I did not want to wear shorts, people kept looking at me and I felt bad. Today I still cannot wear low-cut clothes, because everyone is looking at, asking what it is ... so sometimes I even prefer to walk in full sleeve, in a blouse, so nobody will ask me any question, because if I try to answer, I think I will come across stupid. I also cannot have big beautiful hair, and because I’m always cutting and tidying it, this bothers me a lot. Even finding work is difficult, and that prevents me from being successful professionally. After all, you still have to be under treatment, and I cannot do other things that I like. We often need injections, spending a lot of time here at the clinic. Every day you have to put ointment on instead of doing other things. Psoriasis takes people out of social life, so we go into depression. It's complicated, I'm really depressed, really down.*

### Theme 3 - contributions to improve quality of life in psoriasis

Central ideas:

A. More efficient drugs
B. Clarification of the lay population
C. Acceptance of disease
D. Possibility of healing
E. Treatment adherence
F. Have multidisciplinary service
G. Stress reduction

DCS - *Quality of life could be improved by making other people aware that a person with psoriasis on their skin does not have a disease transmitted by sight, by clothing, by soap, in any way ... Because people stare and are disgusted and scared, but that is a problem that person has. I think that is the reason why a lot of people suffer ... to see the prejudice of others. There comes a time when we stop suffering from these hardships, but for those who are sick at the beginning, they will still have to go through all of this ... Because others look and judge, they are afraid to touch and to be touched. Also, if the treatment advances and they found a cure for it, it would be better, of course ... if you had a medication that would end psoriasis tomorrow, a more direct treatment instead of a weak one. Let's suppose, I'm here today because it's my appointment, but if I did not have to come here, I'd be at work. Now, if I did not have psoriasis, it would be better. It's been a long time since I've had it, that I've lived with it, for about 30 years. With psoriasis, one even forgets what is good. When people do not have this disease it is very good, I still remember how it was... you do not scratch, worry about your appearance, you will be able to be free, going to the beach and pool without thinking. However, I think that this is impossible, that only God can do that at the moment, because for this disease there is no cure. And since this is the reality, what can improve it is to have better control of psoriasis. A control that would minimise the appearance of the lesions on the skin, where there is no more pain. And for this we need to do the right follow-up, more often, because I have a weight problem as well. So, I need an endocrinologist, a nutritionist ... because of the medication, I had side effects in other parts of my body that need to be monitored by other professionals, not just the dermatologist. And not having that makes me a little nervous. This is another thing that would certainly improve quality of life, calm the nerves a bit. It is no use doing the treatment right and not calm down.*

## Discussion

Assessing quality of life in health care settings is challenging, since psychometric instruments can often not accurately translate the magnitude of the impact imposed by any disease on the life of an individual. In the case of psoriasis, this difficulty is contextualized by the chronicity and incurability of a skin disease marked by social restrictions, negative feelings and treatment dependence.

For patients with dermatological diseases, the appearance of their skin plays a fundamental social role, since part of the process of identity development involves appearance. The skin is often the gateway to establishing contact with another person [13], as evidenced in this research. When this does not happen, there is an immense personal discontent in not being able to fulfil the demands and expectations of others, culminating in difficulty in interpersonal relationships and QoL being affected [13].

A study with 41 patients in the UK showed that individuals’ poor understanding and expectations about their illness interfered with their relationships and limited their social interaction [14]. This finding was corroborated in a qualitative study with 16 patients in an outpatient dermatology clinic of a hospital in Denmark, where the stigmatization of the disease led the participants to develop strategies to avoid public exposure and social circumstances [15]. For example, some participants reported to wear clothes that covered their skin lesions to avoid having to explain them. We found the same strategy being reported in our study. A multicentre study with 35 patients showed that the majority suffered social discrimination due to the disease, leading to some individuals lying about it and kept it secret [16].

In the present study, the participants’ accounts highlighted the prejudice and rejection experienced by patients with psoriasis leading to a negative impact on their perceptions of quality of life. Often, this prejudice is so intense that it ends up preventing people not only from social interaction but also from professional fulfilment [17]. Psoriasis also increases levels of psychological stress and body dissatisfaction [17]. The majority of people living with this condition tries to avoid feeling stressed, which is very difficult to be achieved due to the amount of negative thoughts caused by the illness. For the participants, their treatment routine is already a source of stress, since they are prevented from doing things they enjoy.

A study conducted in the United States with 18,000 members of the National Psoriasis Foundation has shown that people with psoriasis believe that physicians quite often underestimate the impact of the disease on patients’ lives. Furthermore, what is perceived by psoriasis patients as severe is often not valued by the physician [18].

Evidence shows that patients prefer an individualised approach to their treatment. They want to be seen as a person, not as a psoriasis case. Some patients report that their complaints are not taken into account by doctors, which hinders their treatment. They report that doctors are not prepared to face the situation and minimize the problem, leaving them with feelings of rejection and with the gravity of their problems underestimated. For them, there is a great need to support the individual issues of their lives beyond illness [15]. This approach is complex and shows that the greatest challenge for professionals providing care for these patients is to see them integrally in an individualized way. It is important to listen and value their complaints and not only focus on the physical aspect of their skin. This study found that, in addition to the medical professional, there is a need and desire to include input from other health professionals, as evidenced in the DCS. This finding concurs with other studies that receiving support from other professionals is highly beneficial [15].

Our findings suggest the importance of having a nursing consultation after an appointment with a specialist. Nurses and specialists could benefit from closer working and collaboration and provide the necessary mutual support. Nursing consultations, follow-ups, integrated health actions and proposed interventions would improve QoL. Interventions should not only be focused on the physical disease. They should be sensitive to the health and social problems of patients seeking positive alternative interventions [19]. A nursing consultation is an effective strategy to improve a patient’s quality of life since it allows a nurse to organize systematically their care aiming at providing care that would involve not only the biological aspect but also an understanding of the patient as a social being during the health-disease process. Such approach would allow a nurse to be part of the process of identifying potential problems and decisions to be made during care [20]. In the present study, having a nurse consultation throughout care has been a positive aspect reported by our patients as feedback. Furthermore, recent evidence shows that having a nurse consultation is beneficial to the community and may result in positive and appropriate measures tailored to the individual care needs of patients [21, 22].

To that end, the literature emphasizes the importance of active listening and attention by professionals, aiming to identify patients’ real needs and then responding with appropriate interventions [23]. Active listening implies discarding pre-defined ideas [24], and requires health professionals’ willingness to change, help and make a difference.

Finally, public health policies to increase knowledge and awareness of the general population about psoriasis are needed. This approach will help to explain the impact of psoriasis on a person’s life and, hopefully, reduce the prejudice surrounding it and facilitate social inclusion. After all, what defines a person goes far beyond their skin.

## Conclusion

The findings from this study reflect not only the perceptions of quality of life, but also reveal the stigma, prejudice and social difficulties faced by patients living with psoriasis. Quality of life for these participants is a subjective, multidimensional concept and often based on the satisfaction of basic human needs for the individual.

The suggestions to improve quality of life, from the perspectives captured by the interviews, were focused on the need to increase awareness of the general population about the disease, the acquisition of more efficient medication that enables healing, acceptance of the disease, care by a multidisciplinary team and stress reduction.

A potential limitation of our study refers to the fact that we did not look into the effects of the study on the participants both during and after the interview and should be included in further studies using our methodology.

Lastly, the present analysis offers important insights to health professionals to improve care strategies through the implementation of actions based on an interdisciplinary care approach, directed to the real psychological and social needs of these patients, which often go unnoticed.

## Supplementary Information

**Additional file 1.** Data collection instrument.pdf

## Data Availability

The data that support the findings of this study are not publicly available because they contain information that compromise the privacy of research participants. However, an anonymous dataset can be made available from the corresponding author under reasonable request.
