# Supplementary material for: Quality of life of patients living with psoriasis: a qualitative study
Source: BMC Dermatol. 2020 Dec 10;20:22. doi: 10.1186/s12895-020-00116-9 (PMC7727128; doi:10.1186/s12895-020-00116-9)
Supplement: Supplementary file 1 — Additional file 1. Data collection instrument.pdf [file 12895_2020_116_MOESM1_ESM.pdf]

Part 1

Clinical and Sociodemographic Information

1. Name: ____________ 2. Phone number: ___________________

3. Date of Birth: ______/______/______ 4. Sex: □ Female □ Male

5. Marital Status: □ Single □ Married □ Divorced/ Widower

6. Education: □ Up to Elementary □ Middle and High school □ Higher education

7. Age of disease onset: ____________

8. Occupation: _______________________

9. Number of people in the family: __________

10. Monthly family income: 1- □ Up to R$ 1,000,00
2- □ R$ 1,100.00 to R$ 3,000.00
3- □ R$ 3,100.00 to R$ 5,000.00
4- □ Over R$ 5,000.00

11. Length of psoriasis treatment: □ less than a year
□ between 5 and 10 years
□ more than 10 years

12. Are you a smoker? □ Yes □ No

13. If yes, how many cigarettes do you smoke per day? __________

14. How long have you been smoking? ________________________

15. If no, how long have you stopped smoking? _________________

16. Do you drink alcohol? □ Yes □ No

17. If yes, how often do you drink? ___________________________

18. If no, when have you stopped drinking alcohol? __________________________

Part 2 - Guided questions applied to patients

1. What do you understand by quality of life?
2. In your opinion, does psoriasis interfere with your quality of life? If yes, in what aspects?
3. In your perception, how could your quality of life be improved in relation to psoriasis?
